# Mechanism of HIFs in osteoarthritis

**DOI:** 10.3389/fimmu.2023.1168799

**Published:** 2023-03-20

**Authors:** Xin-An Zhang, Hui Kong

**Affiliations:** College of Exercise and Health, Shenyang Sport University, Shenyang, China

**Keywords:** osteoarthritis, HIFs, degradation of extracellular matrix, apoptosis, autophagy, inflammatory reaction

## Abstract

Osteoarthritis (OA) is a common disabling disease which has a high incidence rate in the elderly. Studies have found that many factors are involved in the pathogenesis of OA. Hypoxia-inducible factors (HIFs) are core regulators that induce hypoxia genes, repair the cellular oxygen environment, and play an important role in the treatment of OA. For example, HIF-1α can maintain the stability of the articular cartilage matrix, HIF-2α is able to cause chondrocyte apoptosis and intensify in-flammatory response, and HIF-3α may be the target gene of HIF-1α and HIF-2α, thereby playing a negative regulatory role. This review examines the mechanism of HIFs in cartilage extracellular matrix degradation, apoptosis, inflammatory reaction, autophagy and then further expounds on the roles of HIFs in OA, consequently providing theoretical support for the pathogenesis of OA and a new target for OA treatment.

## 1 Introduction

Osteoarthritis (OA) is a common disabling disease that occurs most frequently in the elderly (1). With the growth of the aging population in China, the incidence of OA has showed a rising trend (2). The occurrence of OA is associated with many causes and may be linked to age, gender, obesity, trauma, infection, and other factors (3). OA is a chronic disease, with mild joint swelling and pain in its early stage. With the progression of the disease, the pain becomes increasingly severe, and apparent joint swelling, deformity, and stiffness occur in the late stage (4). In recent years, many studies have confirmed that hypoxia-inducible factors (HIFs) play a vital role in regulating articular cartilage hypoxia; moreover, HIFs are closely related to the progression of OA (5).

HIFs are core regulatory factors that induce hypoxia genes and repair the cellular oxygen environment. HIFs are heterogeneous protein dimers formed by the polymerization of two different subunits: the α and β subunits (6). HIF-1α, HIF-2α, and HIF-3α are three members of the human HIF-α protein family. They are hardly expressed under normal oxygen conditions, but their expressions are significantly up-regulated under hypoxic stress. Under normal oxygen conditions, HIF-1α is rapidly degraded. However, under hypoxia, the HIF-1α degradation is inhibited, its content will increase, and it will enter the nucleus and combine with the corresponding subunits to form an active HIF-1α transcription factor. HIF-1α is mainly involved in regulating autophagy and apoptosis, promoting chondrocyte phenotypes, and maintaining the vitality of chondrocytes (7). In addition, HIF-1α also plays a biphasic role in regulating inflammatory response by regulating VEGF and EPO, and finally promotes chondrocytes to adapt to a hypoxic environment (8). On the contrary, HIF-2α is mainly involved in inducing the expression of catabolic factors in chondrocytes, enhancing the expression of Fas, promoting the apoptosis of chondrocytes, and intensifying the inflammatory reaction, thus leading to the destruction of articular cartilage (9). The functions of HIF-1 α and HIF-2 α are different. Numerous abnormal transformations from HIF-1α to HIF-2α may be one of the mechanisms of OA (10). A moderate reduction in oxygen partial pressure can induce the synthesis of HIF-3α mRNA, and the HIF-3α mRNA level increases with the duration of systemic hypoxia, which appears in many organs, such as lung, kidney and cerebral cortex. The hypoxic induction of HIF-3a depends on HIF-pathway, such as HIF-1 α and HIF-2 α. HIF-3α may be the target gene of HIF-1α and HIF-2α (11, 12). In addition, some transcription factors, such as NFjB, are also involved in the regulation of HIF-3α. NFjB is a transcription factor that is often involved in hypoxia-driven signaling and plays an important role in the regulation of HIF-3α, the inhibition of HIF-3a under hypoxia could be a secondary effect of NFjB inhibition (13).

This review discusses the mechanism of HIFs in the extracellular matrix (ECM) degradation of articular cartilage, apoptosis, inflammatory reaction, and autophagy and then expounds on the roles of HIFs in OA, thereby providing theoretical support for the pathogenesis of OA and identifying a new target for OA treatment.

## 2 Mechanism of pathological changes in osteoarthritis

Pathological changes of OA are mainly reflected in the articular cartilage, subchondral bone and synovial membrane. Articular cartilage degeneration is the earliest pathological change of OA. The main manifestations include softening and cracks in the deep cartilage, with narrowing of the joint space in serious cases (14). The subchondral bone of OA patients also change. If the subchondral bone of OA patients is under excessive pressure, its density will increase, showing ivory sclerosis. In contrast, if the subchondral bone of OA patients is not stimulated by pressure for a long time, the bone density will decrease and eventually shrink. The subchondral bone of OA patients constantly changes with the variations of biological stress, thus forming osteophyte and leading to joint deformities (15). In addition, OA patients generally present with synovitis, mainly caused by the phagocytosis of small cartilage pieces that have fallen into the synovial fluid and joining synovial cells. Congestion and plasma cell infiltration may occur in the early stage, and foreign body giant cell reactions may occur in the later stage (16). These pathological changes are characteristics of OA. Many mechanisms are responsible for these pathological changes, including ECM degradation, apoptosis, inflammatory response, and autophagy.

### 2.1 Degradation of cartilage extracellular matrix

The ECM is a complex network consisting of various macromolecules around cells. Under normal circumstances, the synthesis and metabolism of cartilage extracellular matrix should always keep a dynamic balance. If the ECM of articular cartilage degrades, OA may be induced (17). Metalloproteinase with thrombus motifs, matrix metalloproteinase (MMPs), and Type II collagen are key proteins that induce ECM degradation of articular cartilage and play a key role in maintaining chondrocyte homeostasis (18). Numerous cytokines and non-coding RNA can induce OA by stimulating ECM degradation of articular cartilage. TGF-β is a cytokine which plays an important role in the growth and development of the articular cartilage. TGF-β can stimulate the production of ECM proteins and also block the ECM degradation proteins by increasing protease inhibitor production (19, 20). Therefore, the reduction of TGF-β can induce OA. In addition, many MicroRNAs, such as miR-140 (21), miR-146a (22) and miR-19b (23), can directly regulate chondrocytes and disrupt the balance between the anabolism and catabolism of ECM by affecting their upstream/downstream regulatory factors or pathways. Other studies have shown that chondrocytes will undergo different changes in metabolism after encountering external mechanical stimuli such as hypoxia (Abramson and Attur, 2009), which will eventually lead to the degradation of ECM (24).

### 2.2 Apoptosis

Many stimuli can cause apoptosis of chondrocytes, and a correlation exists between the damage degree of chondrocytes and apoptosis, and obvious apoptosis occurs in the cartilage of OA patients (25). On the one hand, OA cartilage can produce considerable nitric oxide (NO). Low-level reactive oxygen species (ROS) can induce apoptosis in the presence of NO, and high-level ROS can cause necrosis. On the other hand, mitochondria play a crucial role in cell function and survival, and the damage to the outer membrane of the mitochondria in OA chondrocytes will cause the release of apoptosis factors such as caspase-8 and caspase-9 into the cytoplasm from the gap of the mitochondrial membrane, eventually leading to cell apoptosis (26). Studies have proved that hypoxia can stimulate the generation of ROS in mitochondria of chondrocytes and induce apoptosis (7). In addition, various physical or chemical stimuli can regulate OA through biomarkers or signal pathways such as Bcl-2, Bax, JNK and MAPK that affect apoptosis. For example, Kong et al. (27) found that mechanical stimulation can promote the phosphorylation of JNK and MAPK and regulate the apoptosis of chondrocytes.

### 2.3 Autophagy

Furthermore, autophagy is closely related to apoptosis and can realize the metabolic needs of cells and the renewal of some organelles. In the early stage of OA, autophagy is activated to avoid apoptosis. In the late stage of OA, apoptosis increases extensively and can be activated at the same time as apoptosis (28). Numerous biomarkers, such as Beclin1, LC3, and P62, are related to autophagy. Takayama et al. (29) established an OA mouse model and treated the subjects with rapamycin. The results showed that the expression of mTOR decreased, LC3 was activated, and the severity of OA was reduced in rapamycin-treated mice. When oxidative stress occurs, cell autophagy will also be activated (30), and AMPK can directly regulate autophagy by acting on the downstream signal molecules of mTOR (31), an occurrence which ultimately affects the pathological process of OA. Studies have proved that oxidative stress and hypoxia can stimulate mitochondrial autophagy and promote cell survival (7).

### 2.4 Inflammatory reaction

Inflammation is the body’s response to infection, pathogenic microorganisms, trauma, allergy, and other tissues and cells and is also the body’s defensive response (32). For patients in the late stage of OA, pro-inflammatory cytokines and chemokines in synovial fluid increase (33), which could increase the expression of collagenase and aggrecan, induce the inflammatory reaction, and lead to the ECM degradation of articular cartilage (34). TNF, IL-1β, IL-6, and IL-15 are cytokines related to inflammation, and NF-κB, MAPK, and AMPK are common signal pathways to induce the inflammatory response. Some studies have found that regulating NF-κB/SIRT1/AMPK and MAPK/NF-κB signaling pathways can affect the inflammatory response and ultimately affect the pathological process of OA (35) (36). In addition, Hypoxia can also aggravate inflammatory reaction by affecting macrophages, T cells and neutrophils (37). Therefore, the changes of some cytokines and inflammation-related signaling pathways, or the hypoxia environment can aggravate the inflammatory reaction and ultimately affect the pathological process of OA.

**Figure 1:**
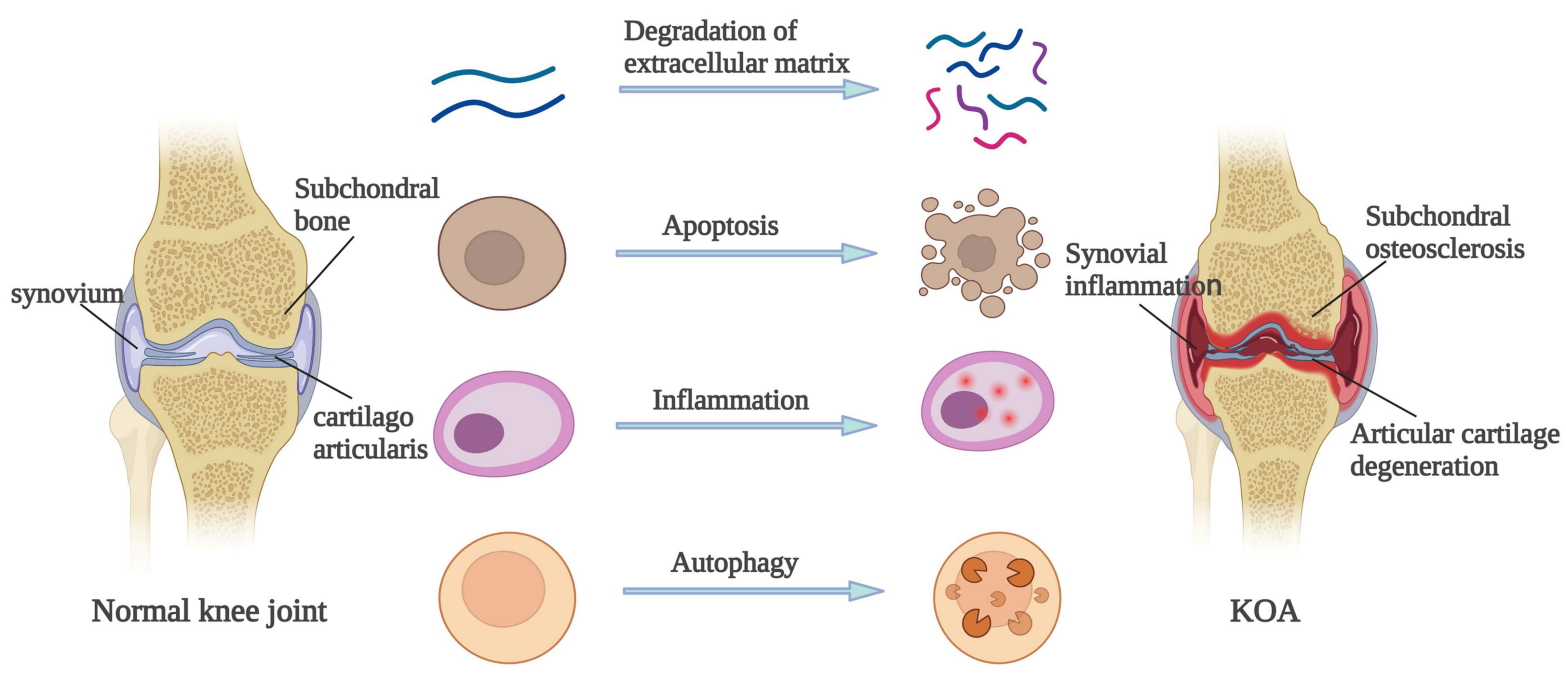
Pathological change mechanism of OA.

In conclusion, the pathological changes of the articular cartilage, subchondral bone, and synovium in OA patients are generally caused by ECM degradation, apoptosis, autophagy and inflammatory reaction. As shown in **Figure 1**.

## 3 HIFs in pathological mechanism of osteoarthritis

**Figure 2:**
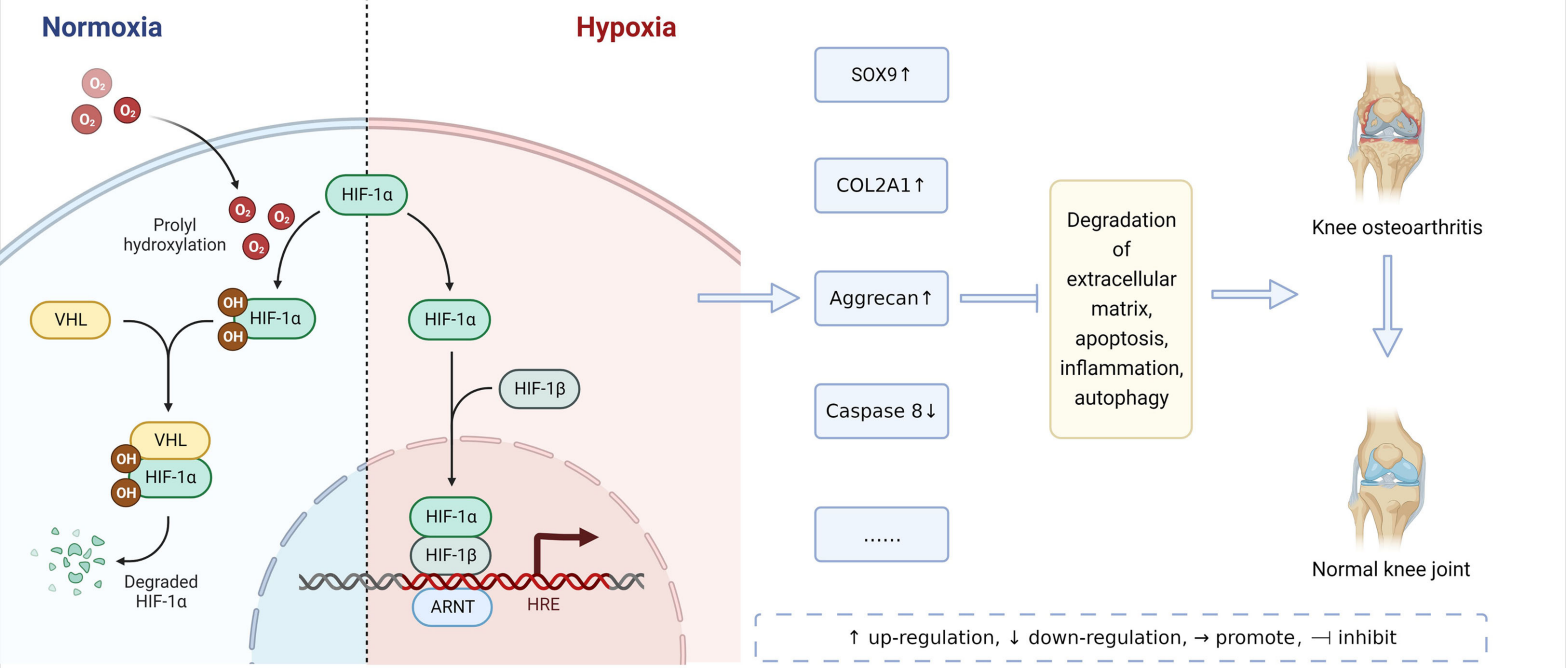
Mechanism of HIFs on OA in Normoxic and Hypoxic Environments.

HIFs have different mechanisms in normoxic and hypoxic environments, and they can alleviate the pathological process of osteoarthritis, as shown in **Figure 2**. Further, HIFs are closely related to the pathological mechanism of OA. Bouaziz et al. (38) and Yang et al. (39) proved the role of HIF-1-α and HIF-2-α in OA for the first time by using the mouse model of functional loss and functional gain respectively. HIFs and HIFs-related genes and signal pathways can participate in the pathological process of OA by affecting ECM degradation, apoptosis, autophagy, and inflammatory response. as shown in **Table 1**.

**Table 1:** Functional characterization of the HIFs in OA.

Model	HIFs	Related gene/cytokines/protein	Involved in pathways	ncRNA	mechanism of action	References
OA mouse model	HIF-1α	/	/	/	mitophagy	(7)
OA mouse model	HIF-1α	Traf6、IRAK1、Bcl-2	/	microRNA-146a	autophagy	(40)
OA rat model	HIF- 1α	SOX9	/	/	cartilage degeneration	(41)
OA rat model	HIF- 1α	NLRP1、NLRP3	/	/	synovial fibrosis	(42)
OA rat model	HIF-1 α	Agnuside、NLRP3	/	/	Synovitis and Fibrosis	(43)
OA rat model	HIF-1α	JAK2	miR-216a-5p/JAK2/STAT3	miR-216a-5p	Proliferation, Migration and Apoptosis of Cartilage Cells	(44)
OA cell model	HIF-1α	/	/	/	Cartilage cell viability	(45)
OA cell model	HIF-1α	PKM2	/	/	Cell proliferation and apoptosis	(46)
OA cell model	HIF-1α	MMP1	/	/	Cartilage destruction	(47)
OA mouse model	HIF1α	Bmal1	HIF1α-VEGF	/	Cell proliferation and apoptosis	(48)
OA mouse model	HIF-1α	MMP3、MMP13、ADAMTS5 、NOS2	/	MiR-17	Cartilage homeostasis	(49)
OA rabbis model	HIF- 1α	DHJST、VEGF	/	/	apoptosis	(50)
OA rat model	HiF-1α	Vegf、Opg	/	/	Osteoclast formation	(51)
OA rat model	HIF-1α	CD44 、HA	/	/	Synthesis of extracellular matrix	(52)
OA cell model	HIF-1α	IL-1β、IGF-I、IGF-II	PI-3K	/	Cartilage homeostasis	(53)
OA cell model	HIF-1α	HGF、c-Met 、 VEGF-A	c-Met/PI3K/Akt 、mTORC1	/	Cartilage homeostasis	(54)
OA mouse model	HIF-1α	/	/	/	Synthesis and decomposition of cartilage	(55)
OA mouse model	HIF-1α	CRAT	/	miR-144-3p	apoptosis	(56)
OA cell model	HIF-1α	VEGF、BNIP3	PI3K/AKT/mTOR	LncHIFCAR	apoptosis	(57)
OA cell model	HIF-1α	/	/	/	Autophagy	(58)
OA rat model	HIF-1α	Aggrecan、ADAM-TS5	/	/	Cartilage homeostasis	(59)
OA cell model	HIF-1α	/	/	CircRNA-UBE2G1 、miR-373	Cell proliferation, apoptosis and synthesis of extracellular matrix	(60)
OA rat model	HIF-1α	HMGB1、 Erk、 JNK	/	/	Inflammationsynovial angiogenesis、	(61)
OA mouse model	HIF-1α	C1qtnf3	NF-κB	/	Resist the catabolism of cartilage	(62)
OA cell model	HIF-1α	VEGF	PI3K/AKT	/	Cartilage degeneration	(63)
OA rat model	HIF -1α	Icariin	TDP‐43	/	apoptosis	(64)
OA mouse model	HIF-1α	VEGF	/	/	Articular cartilage homeostasis	(65)
OA rat model	HIF-1α	Casticin	HIF-1α/NLRP3	/	Synovitis and fibrosis	(66)
OA mouse model	HIF-1α	Baicalin	/	/	Synthesis of extracellular matrix	(67)
OA rat model	HIF-1α	Vitexin	/	/	inflammation	(68) pathway
OA mouse model	HIF-1α	Bcl-2	/	MicroRNA-146a	Autophagy	(69)
OA rat model	HIF-1α	GLUT1	HIF-1α-GLUT1	/	Cartilage cell degradation	(70)
OA cell model	HIF-1α	//	/	miRNA-411	Autophagy	(71)
OA cell model	HIF-1	PRP	/	/	Autophagy and apoptosis	(72)
OA rat model	HIF-1	VEGF、Notch	HIF-1-Notch-VEGF	/	angiogenesis of condylar cartilage	(8)
OA cell model	HIF-1	PRP	/	/	Joint pain and stiffness	(73)
OA mouse model	HIF-2α	D-mannose、Gpx4、Scl7a11	/	/	chondrocyte ferroptotic cell death	(74)
OA mouse model	HIF-2α	Sox9	/	miR-455-3p、miR-455-5p	regulate articular cartilage homeostasis	(75)
OA mouse model	HIF-2α	MIA、COX-2、 RUNX2	NF-κB	/	cartilage degradation、inflammation	(76)
OA rat model	HIF-2α	CMC2.24	NF-κB	/	Cartilage homeostasis	(77)
OA rat model	HIF-2α	MMP13、ADAMTs-4	NF-κB	/	Cartilage degradation	(78)
OA mouse model	HIF-2α	ZIP8、MTF1	/	/	cartilage destruction	(79)
OA mouse model	HIF-2α	AKP	/	/	apoptosis	(80)
OA mouse model	HIF-2α	CJM	/	/	Cartilage destruction、inflammation	(81)
OA rat model	HIF-2α	/	/	/	Inflammation、hypoxia	(82)
OA mouse model	HIF-2α	SIRT1	/	/	Decomposition of cartilage	(83)
OA rat model	HIF-2α	PRP	/	/	Chondrocyte apoptosis and inflammation	(84)
OA mouse model	HIF-2α	AURKA、NEDD9	HIF2α/AURKA/NEDD9	/	mediating the primary cilia loss	(85)
OA mouse model	HIF-2α	syndecan-4	/	miR-96-5p	Cartilage degradation	(86)
OA rat model	HIF-2α	ChM-1	/	/	Cartilage homeostasis	(87)
OA mouse model	HIF-2α	Wogonoside	PI3K/AKT、 NF-κB/HIF-2α	/	Degradation of extracellular matrix and chondrocyte hypertrophy	(88)
OA cell model	HIF-2α	OPN、CD44	/	/	Cartilage cell homeostasis	(89)
OA mouse model	HIF-2α	Curcumin	NF-κB/HIF-2α	/	inflammation	(90)
OA rat model	HIF -2α	YH23537	/	/	Pain, cartilage degeneration	(91)
OA mouse model	HIF-2α	IKK、BMS-345541	NF-κB-HIF-2α	/	Synthesis and decomposition of cartilage	(92)
OA mouse model	HIF-2α	MitA	NF-κB-HIF-2α	/	Cartilage cell catabolism	(93)
OA cell model	HIF-2α	/	/	MicroRNA-365	The catabolism of cartilage	(94)
OA rat model	HIF-2α	Rebamipide	/	/	Oxidative stress、Cartilage homeostasis	(95)
OA mouse model	HIF-2 α	/	/	/	apoptosis	(9)
OA mouse model	HIF-2 α	Atrogin-1	/	/	The catabolism of cartilage	(96)
OA mouse model	HIF-2α	Nampt	/	/	The catabolism of cartilage	(97)
OA cell model	HIF-2α	COL10、MMP13	/	/	apoptosis	(98)
OA cell model	HiF-2α	Leptin、DKK2	/	/	Bone remodeling	(99)
OA mouse model	HIF- 2α	NAMPT	/	/	Degradation of extracellular matrix	(100)
OA cell model	HIF-2α	IPFP	/	/	chondrogenesis	(101)
OA cell model	HIF-1α, HIF-2α	NDRG3	/	/	Synthesis and catabolism of cartilage	(102)
OA mouse model	HIF-1α, HIF-2 α	resveratrol	AMPK/mTOR	/	autophagy	(103)
OA mouse model	HIF-1α, HIF-2 α	Vhl	/	/	Apoptosis, Autophagy and Cartilage Matrix Decomposition	(104)
OA cell model	HIF-3α	COL2A1、COL10A1、MMP13	/	microRNA-210	cell proliferation	(105)
OA rat model	HIF-3 α	/	/	MicroRNA-210-3p	chondrogenesis	(106)

### 3.1 HIFs in the degradation of cartilage extracellular matrix

HIFs may be involved in the ECM degradation of articular cartilage. In a hypoxic environment, chondrocytes can make an adaptive response to glycolysis through the HIF-α transcription factor. HIF-1α and HIF-2α are mainly expressed in chondrocytes. HIF-1α promotes cartilage homeostasis by maintaining anaerobic glycolysis, stabilizing articular cartilage phenotype, inducing proper autophagy, and inhibiting ECM degradation of articular cartilage (5). In OA cells, the increased HIF-1α expression can promote the increase of ECM of articular cartilage (67). For example, Kovács et al. (107) confirmed that when OA occurred, depriving the chondrocytes of oxygen could activate HIF-1α and promote ECM of articular cartilage synthesis. Hu et al. (7) established an OA mouse model and detected the levels of the HIF-1α gene and protein *in vivo* and *in vitro* experiments. They found that HIF-1α expression was up-regulated in human and mouse knee joint cartilage. HIF-1α can inhibit ECM degradation of articular cartilage by mediating mitochondrial autophagy, thereby alleviating the metabolic imbalance of ECM and finally clearing OA. However, HIF-2α is a catabolic transcription factor. It is involved in the formation of chondrocyte phenotype induced by hypoxia but has the effect of antagonizing part of HIF-1α. In OA, HIF-2α expression in articular cartilage increases, which encourages the catabolic reaction by promoting chondrocyte hypertrophy and differentiation and increasing the expression of MMP family-related factors (108).

Many studies have found that we can improve the ECM by regulating HIFs through some small molecules. Some protein molecules can regulate HIF-1α and participate in ECM degradation of articular cartilage. For example, pyruvate kinase M2(PKM2) can handle HIF-1α. Low PKM2 knockdown inhibits HIF-1α, which reduces the expression of COL2A1 and SOX-9 and ultimately accelerates the ECM degradation of articular cartilage (46). Some compounds can also regulate HIF-1α expression. For example, Icariin can increase HIF-1α expression, promote anaerobic glycolysis metabolism, increase cell vitality, and promote ECM of articular cartilage production (109). Both wogonin (88) and curcumin (77) can regulate ECM of articular cartilage homeostasis by inhibiting the NF-κB/HIF-2α axis and thus reduce OA progression.

A variety of non-coding RNAs can be involved in the ECM degradation of articular cartilage by mediating HIFs to promote the expression of related genes. Hwang et al. (94) studied human chondrocytes of OA patients. They found that compared with normal cartilage, human OA cartilage had a significantly decreased miR-365 level, increased HIF-2α mRNA level, and significantly increased level of HIF-2α positive cells. Therefore, the decreased expression level of miR-365 in OA can up-regulate the expression of HIF-2α, increase the expression of a variety of catabolic genes, and ultimately promote ECM degradation of articular cartilage. In addition, miR-455 expression is decreased in OA cartilage. MiR-455s can inhibit the expression of HIF-2α and increase the expression of genes related to cartilage degradation (75). HIF-3α also plays a role in regulating the ECM of articular cartilage. Li et al. (105) noted reduced expression of miR-210 in OA cartilage by reverse transcription-polymerase chain reaction (RT-PCR). Through the Western blot (WB) analysis, they further found that miR-210 overexpression could inhibit the mRNA and protein expression level of HIF-3α in OA chondrocytes. When the expression level of HIF-3α is decreased, the mRNA level of COL2A1 is increased and the mRNA levels of MMP13 are reduced, resulting in increased ECM deposition in OA chondrocytes. Therefore, some cytokines, small molecular compounds and non-coding RNA can affect the ECM degradation of articular cartilage by regulating HIFs.

### 3.2 HIFs in apoptosis of cartilage and bone

Some HIFs participate in the occurrence and development of OA by mediating apoptosis. Different HIFs subtypes have different effects on chondrocyte apoptosis in a hypoxic environment. For example, HIF-1α can inhibit chondrocyte apoptosis, while HIF-2α can promote chondrocyte apoptosis in the hypoxic environment. It is necessary to detect the expression of HIFs in degenerative articular cartilage. Huang et al. (110) intervened in chondrocytes with hypoxia and found that the expressions of HIF-1α and HIF-2α were up-regulated, and the apoptosis rate of chondrocytes increased. In chondrocytes and tissues, HIF-3α is negatively correlated with hypertrophy markers COL10A1 and MMP13. In OA chondrocytes, HIF-3α expression is lower than that of healthy chondrocytes, and MMP13 expression is higher, resulting in a high apoptosis rate (111). In a mouse model of OA induced by mechanical stress, researchers found that the expression of HIF-1α decreased and the expression of caspase 3 increased in the chondrocytes of OA mice and concluded that the HIF-1α signal might participate in chondrocyte apoptosis by inhibiting the caspase cascade and slowing down the process of OA (65).

In addition to a hypoxic environment, many cytokines and growth factors can stabilize and activate HIF-1α, such as the chondrocyte catabolism factors IL-1β and TNF-α. Stimulation of cultured synovial fibroblasts with IL-1β and TNF-α increase HIF-1α mRNA levels. The enhancement of HIF-1α activity can promote energy production and cartilage matrix protein synthesis in OA chondrocytes under hypoxia and can also enhance the expression of anti-apoptosis factors (45). Therefore, many cytokines, protein molecules, and small molecular compounds can accelerate or inhibit apoptosis by regulating HIFs, thereby exerting a certain impact on OA. For example, through immunohistochemistry, WB, and RT-PCR, Ma et al. (48) discovered that the increased Bmal1 expression in mouse Osteoarthritis chondrocytes could up-regulate HIF1α and HIF2α expression, inhibit the levels of MMP13 mRNA and protein, impede apoptosis, and alleviate the process of OA. Yang et al. (84) measured the mRNA and protein expression levels of HIF-2α in mouse OA chondrocytes and confirmed that HIF-2α was up-regulated in OA chondrocytes compared with that of normal cells. Platelet-rich plasma (PRP) inhibits the activation of HIF-2α by reducing the expression of apoptotic markers such as MMP3 and MMP13. Therefore, PRP can reduce the IL-1β-induced apoptosis of chondrocytes by inhibiting HIF-2α. Ryu et al. (9) noted significantly increased levels of HIF-2α in human and mouse OA chondrocytes. Overexpression or knock-down of HIF-2α alone does not induce apoptosis in chondrocytes. However, HIF-2α expression significantly increases chondrocyte apoptosis in the presence of agnostic anti-Fas antibody. Thus, HIF-2α can enhance Fas-mediated apoptosis of chondrocytes and aggravate the pathological process of OA.

Activation of HIF-related signaling pathways may also accelerate or inhibit apoptosis. Using an OA mouse model, Rong et al. (44) found that miR-216a-5p expression was increased in small extracellular vesicles and then gradually transferred to chondrocytes. JAK2 is a target gene of miR-216a-5p. Through the miR -216a-5p/JAK 2/STAT 3 signaling pathway, HIF-1α can induce hypoxic bone marrow mesenchymal stem cells to release small extracellular vesicles and inhibit the apoptosis of chondrocytes. In addition, OA chondrocytes can promote the migration of vascular endothelial cells under the stimulation of TNF-α by secreting chemokines and vascular endothelial growth factors. The invasion of vascular endothelial cells leads to an increase in oxygen tension in the local environment, which activates the JAK-STAT5 pathway. Moreover, the binding of phosphorylated STAT5 to specific sites in the SED7 promoter increases the transcription of SED7. SED7 mediates chondrocyte apoptosis by inhibiting the nuclear localization of HIF-1α and participates in the occurrence and development of OA (112).

Some non-coding RNAs are also involved in apoptosis by mediating HIFs. Song et al. (113) found that in the articular chondrocytes of mice knocked out by HIF-1α, the expression level of CRAT was decreased and that of miR-144-3p was increased. Through further observation and analysis of the articular chondrocytes, they confirmed an increase in the apoptosis of chondrocytes. Therefore, HIF-1α overexpression can stimulate CRAT expression and inhibit miR-144-3p expression, thereby inhibiting apoptosis and ultimately alleviating OA. VEGF and BNIP3 are target genes of HIF-1α. LncHIFCAR promotes hypoxia-induced inflammatory response and matrix synthesis by upregulating VEGF and induces apoptosis by upregulating BNIP3. LncHIFCAR is up-regulated in OA tissues, and LncHIFCAR inhibition may improve hypoxia-induced apoptosis and cell damage and alleviate OA progression by positively regulating HIF-1α and HIF-1α target genes (VEGF and BNIP3) (57). Chen et al. (60) took human chondrocytes and induced an OA chondrocyte model by LPS. They found significantly increased expression levels of circRNA-UBE2G1 and HIF-1α in OA tissues and down-regulated expression levels of miR-373. CircRNA-UBE2G1 binds to miR-373 as competitive endogenous RNA (ceRNA). HIF-1α may also be a target for miR-373. Therefore, circRNA-UBE2G1 can induce apoptosis and ultimately accelerate the OA progression by regulating the miR-373/HIF-1α axis. Therefore, some cytokines, small molecular compounds and non-coding RNA can affect the apoptosis by regulating HIFs.

### 3.3 HIFs in inflammatory response

HIFs are closely related to the inflammatory response in OA. Many genes, proteins, and compounds can participate in the inflammatory response by regulating HIF-1α. Under hypoxic conditions, HIF-1α expression is up-regulated in OA synovial fibroblasts, leading to inflammatory cell recruitment and angiogenesis (114). High-mobility group 1 protein (HMGB1) is a protein associated with inflammation. Feng et al. (61) stimulated rat synovial fibroblasts with HMGB1 to investigate the expression of VEGF and HIF-1α in these cells by using WB, RT-PCR, and immunofluorescence. They verified that inflammatory factors such as IL-6, IL-1β, and TNF-α were increased in rat synovial fluid. Moreover, by activating Erk and JNK, HMGB1 up-regulated VEGF and HIF-1α in OA synovial fibroblasts to participate in the inflammatory response. Some compounds can affect inflammatory response by regulating HIFs. The expression of HIF-1α, NLRP3, and IL-1β increases in the absence of oxygen. Imperatorin (IMP) can alleviate synovitis and synovial fibrosis and improve the symptoms of OA by inhibiting HIF-1α/NLRP3 inflammasome signaling (115). Agnuside ameliorated the hypoxia in KOA rats and inhibited the accumulation of HIF-1 α and activation of NLRP3 inflammasome in LPS-treated fibroblast-like synovial cells, thereby alleviating synovitis in KOA rats and alleviating the pathological conditions of OA (43). Li et al. (66) established an OA rat model, intervened with casticin, and detected the inflammatory components and the expression of HIF-1α by using WB, RT-PCR, or ELISA. The results showed that capsaicin inhibited the MIA-induced activation of NLRP3 inflammasomes in OA rats and synovial fibroblasts, inhibited the expression of HIF-1α, and reduced the hypoxia and inflammation of synovial tissue in rats, finally alleviating OA.

In addition, proper exercise and some compounds can also affect the inflammatory response by regulating HIF-2α. Wang et al. (116) established an OA rat model and applied whole-body shaking exercises at different frequencies to detect the expression of relevant genes and proteins by using RT-PCR and WB. They found that in the early OA knee joint cartilage, whole-body vibration training could reduce the levels of inflammatory factors, inhibit HIF-2α expression, and alleviate the pathological changes of the OA cartilage. Through *in vivo* and *in vitro* experiments, Cho et al. (81) demonstrated that apigenin could effectively reduce HiF-2α expression and inhibit the manifestations of IL-6 and COX-2 in HIF-2α-induced articular chondrocytes, thereby reducing inflammation and relieving OA. Therefore, many compounds can slow down the inflammatory reaction by regulating different subtypes of HIFs.

### 3.4 HIFs in autophagy

HIFs are closely related to autophagy. Lu et al. (58) took human OA chondrocytes induced by IL-1β and conducted an *in vitro* experiment. They found high basal autophagy levels in chondrocytes in an environment with elevated HIF-1α, and the chondrocytes were resistant to IL-1β-induced inflammatory damage. Some special environments and compounds can participate in autophagy by regulating HIFs. Moussa et al. (72) cultured human OA chondrocytes in PRP. RT-PCR and ELISA detected the expressions of autophagy markers such as BECLIN, LC3II, and HIF-1. They finally found that the terms of autophagy markers BECLIN and LC3II were up-regulated, as well as the expression of HIF-1 mRNA. Therefore, PRP can participate in cartilage protection and alleviate OA by up-regulating HIF-1 and promoting autophagy. Qin et al. (103) established an OA mouse model and judged the effect of resveratrol on autophagy and HIF expression by injecting resveratrol into the joint cavity. They confirmed that resveratrol delayed articular cartilage degeneration and promoted chondrocyte autophagy, which could balance the expression of HIF-1α and HIF-2α and ultimately regulate the AMPK/mTOR signaling pathway to protect articular cartilage.

Some non-coding RNA can also be involved in autophagy by mediating HIFs. MiR-146a and HIF-1α expression were up-regulated when the chondrocytes of OA mice were exposed to hypoxia. RT-PCR and WB revealed that miR-146a could induce HIF-1α expression in hypoxia and then promote autophagy by reducing the expression of autophagy inhibitor Bcl-2 and finally alleviate OA (40, 69). By TargetScan analysis, Yang et al. (71) experimented with human OA chondrocytes and revealed that HIF-1α mRNA was the direct target of miR-411. They further verified that miR-411 could directly recognize the predicted HIF-1α mRNA site and inhibit HIF-1α expression in chondrocytes through luciferase reporter gene detection. Therefore, miR-411 promotes chondrocyte autophagy by targeting HIF-1α. Therefore, some special environments, compounds or non-coding RNA can participate in autophagy by regulating HIFs.

## 4 Conclusion and prospect

Hypoxia and HIFs participate in a series of pathophysiological processes such as cartilage destruction, synovial inflammation, and angiogenesis in OA. Therefore, HIFs may be closely related to the pathological mechanism of OA. With increasing in-depth research on HIFs, their regulatory role in cartilage physiology and pathology gradually becomes clear, and HIFs can participate in various signal pathways to regulate the survival and metabolism of chondrocytes. HIFs can affect the pathological changes of articular cartilage, subchondral bone, and synovium by participating in ECM degradation of articular cartilage, apoptosis, inflammatory reaction, and autophagy. Moreover, HIFs play an essential role in the pathological changes of OA.

Although a growing number of studies have been conducted on the metabolism, growth, and apoptosis of articular cartilage, the study on the role of HIFs in disease regulation, diagnosis and treatment remains in the early stage (117), and many problems need to be solved urgently. First, under different oxygen concentrations, different levels of chondrocytes have distinct responses to oxygen concentrations, so researchers should explore appropriate oxygen concentrations to promote cartilage repair at different levels (118). Second, cartilage repair is a complex process influenced by many factors, so further study on the combination of HIFs and other growth factors need to be made to promote cartilage repair (71). Moreover, the effects of different subtypes of HIFs on chondrocytes vary in a hypoxic environment and the role of each subtype in cartilage repair should be further explored. Finally, the functions of HIFs in different stages of OA may be different, and the subtypes of HIFs should be selectively used to delay the progression of OA in each stage of the disease (119).

As for the future, with the role of different oxygen concentrations, subtypes of HIFs, and combined use of various growth factors in cartilage repair becoming increasingly clear, researchers can consider HIFs as the therapeutic target of OA, an approach which will achieve further breakthroughs and progress in OA treatment. This review summarizes the role of HIFs in ECM degradation, apoptosis, inflammation, and autophagy of OA and expounds on the current research progress of HIFs in the prevention, diagnosis, and treatment of OA, thereby providing a basis for the future treatment of OA with HIFs.

## Author contributions

X-AZ: conceptualization, project administration, and funding acquisition. HK and X-AZ: writing – review and editing. All authors contributed to the article and approved the submitted version. HK and X-AZ contributed equally to the article and should be regarded as co-first authors.
